# Supplementary material for: Meaning in life, positive cognition, and learning motivation: A mediational analysis among Chinese college students
Source: PLoS One. 2025 Sep 12;20(9):e0330447. doi: 10.1371/journal.pone.0330447 (PMC12431252; doi:10.1371/journal.pone.0330447)
Supplement: S2 Appendix — (PDF) [file pone.0330447.s002.pdf]

1. The Meaning in Life Questionnaire (MLQ) - Chinese Version

Authors: Original scale by Steger, M. F., Frazier, P., Oishi, S., & Kaler, M. (2006). Revised Chinese version by Liu, S., & Gan, Y.

Description: This questionnaire has 9 items, containing two dimensions: Presence of Meaning (MLQ-P) and Search for Meaning (MLQ-S). MLQ-P includes items 2, 4, 7, 8, 9; MLQ-S includes items 1, 3, 5, 6. The scale uses a 7-point Likert scale, from 1 (Absolutely Untrue) to 7 (Absolutely True).

Source: Liu, S., & Gan, Y. (2010). Reliability and validity of the Chinese version of the Meaning in Life Questionnaire in college students. *Chinese Journal of Clinical Psychology, 24*(6), 478-482.

Items:

1. I am searching for a purpose or mission for my life. (MLQ-S)
2. My life has no clear purpose. (MLQ-P, reverse scored)
3. I am searching for meaning in my life. (MLQ-S)
4. I understand my life's meaning. (MLQ-P)
5. I am looking for something that makes my life feel meaningful. (MLQ-S)
6. I am always trying to find my life's purpose. (MLQ-S)
7. My life has a clear sense of direction. (MLQ-P)
8. I know what makes my life meaningful. (MLQ-P)
9. I have discovered a satisfying life purpose. (MLQ-P)

---

2. Learning Motivation Questionnaire (LMQ)

Author: Compiled by Wang, W.↳

Description: This questionnaire consists of 16 items, divided into two independent dimensions: intrinsic motivation and extrinsic motivation. It uses a 5-point Likert scale, from 1 (Completely Disagree) to 5 (Completely Agree).

Source: Wang, W. (2006). *A Comparative Study of Self-Efficacy, Learning Motivation, and Achievement Goal Orientation Between High-Achieving and Underachieving College Students* [Master's thesis, Nanjing Normal University].

Items:

1. During my studies, I often work extra hard to meet the academic standards my parents expect of me.
2. When I successfully complete a learning task, I feel a sense of accomplishment, even if no one else knows.
3. I believe that studying hard is a means to achieve a comfortable life.
4. In order to develop professionally, I will do my best no matter how difficult the study is, as long as I think it's worthwhile.
5. I strive to obtain things that are generally considered valuable for career prospects, such as professional certificates or passing the College English Test Band 6.
6. I pursue a higher degree not to bring honor to my family, but because I have a personal interest in knowledge.
7. My main goal in studying is to gain the approval of others.
8. When I study, I always try my best to prove my ability.
9. I think it is very important to master the knowledge of my major.
10. I have to study in order to pass exams and get my diploma.
11. Only by studying hard and getting good grades can I be qualified for a good job.
12. I believe that mastering professional knowledge will be of great help in my future career.
13. I am very interested in the knowledge of my major.
14. I study hard to enrich myself and improve my own abilities.
15. I must study hard to repay my parents and allow them to live a better life.
16. I believe that through learning, I can continuously acquire new knowledge and improve my personal qualities.

---

3. The Attention to Positive and Negative Information scale (APNI) - Chinese Version

Note: Only the items for the Attention to Positive Information (API) subscale were used in the study and are provided below.

Authors: Original scale by Noguchi, K., Gili, P., & Sakamoto, S. (2014). Revised Chinese version by Dai, Q., & Feng, Z.

Description: The full scale contains 22 items, divided into the Attention to Positive Information (API) subscale and the Attention to Negative Information (ANI) subscale. This study utilized the API subscale. The scale uses a 5-point Likert scale, from 1 (Completely Disagree) to 5 (Completely Agree).

Source: Dai, Q., Feng, Z., Xu, S., Xie, J., Liu, K., Yu, Y., et al. (2015). Validity and reliability of the Chinese Attention to Positive and Negative Inventory in college students. *Chinese Mental Health Journal*, 29(5), 395-400.

Items (API Subscale):

1. I notice good things as much as other people do.
2. I pay special attention to the many small pleasures that life brings me.
3. I notice things that make me feel inspired.
4. There are many things in my life that I enjoy.
5. I pay special attention to the personality traits that my family and friends praise in me.
6. I focus on the good parts of my character.
7. For me, it is important to remember the good things about others.
8. I want to improve myself in many ways.
9. No matter who is laughing, I notice happy faces.
10. I notice and focus on the moments when everything is going well.
11. I pay special attention to the things I have done successfully.
12. I can easily see the fun side of all the activities I participate in.
