# Supplementary figures and images for: Meaning in life, positive cognition, and learning motivation: A mediational analysis among Chinese college students
Source: PLoS One. 2025 Sep 12;20(9):e0330447. doi: 10.1371/journal.pone.0330447 (PMC12431252; doi:10.1371/journal.pone.0330447)

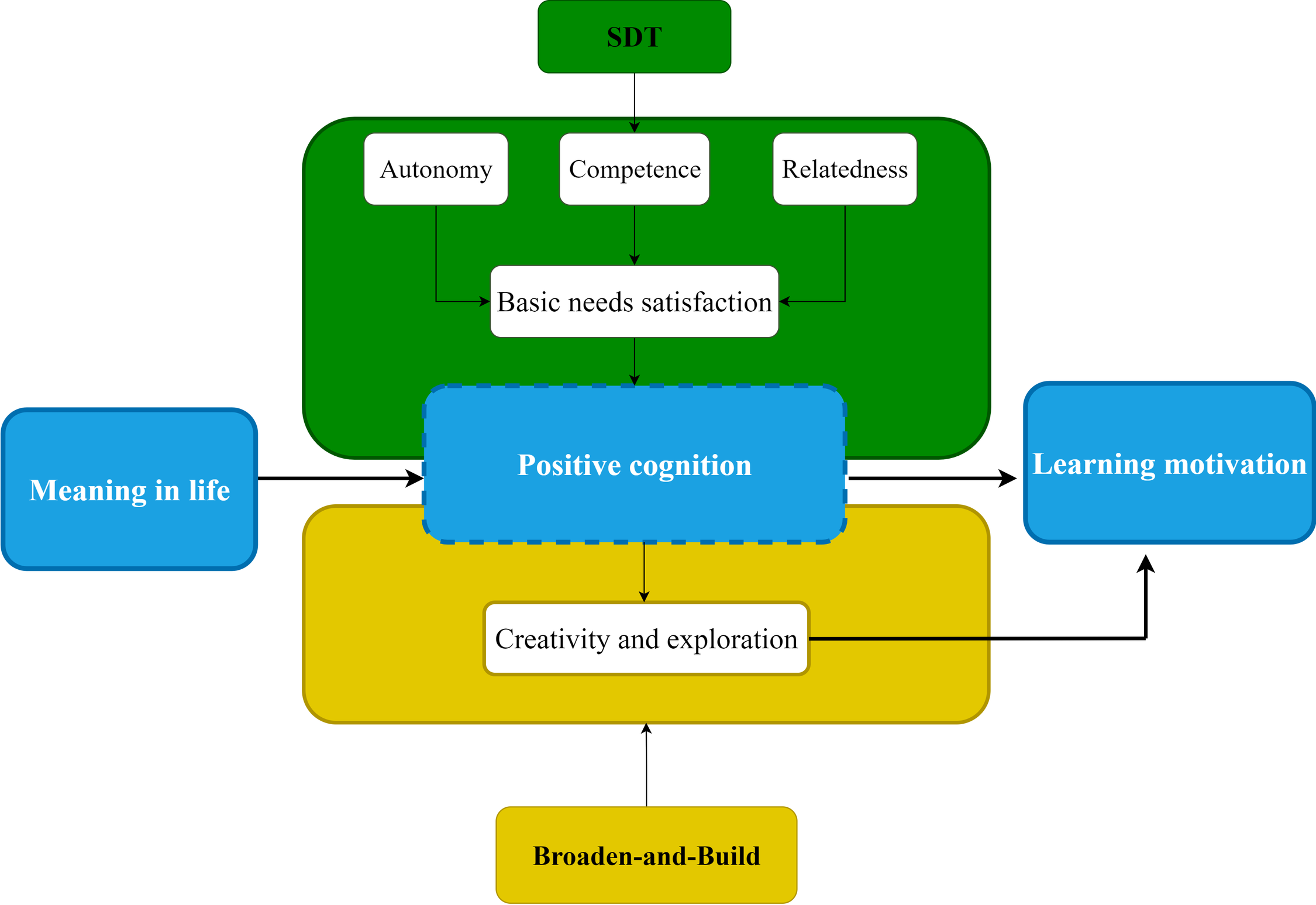

Supplement: S1 Fig — (TIF) [file pone.0330447.s003.tif]

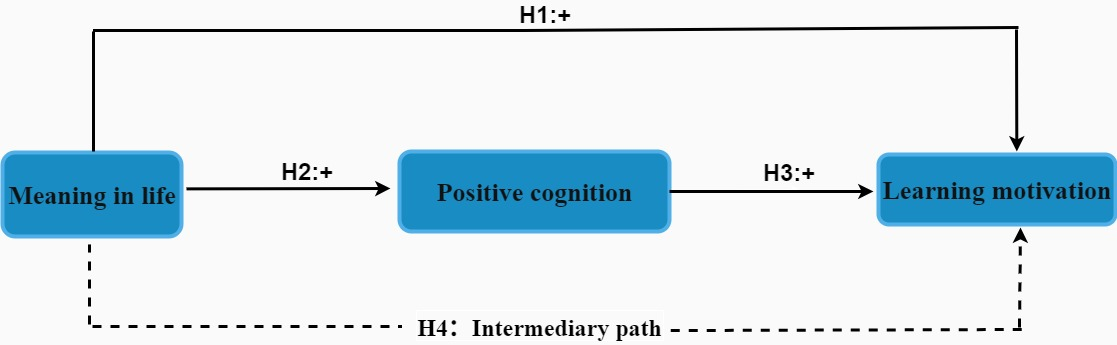

Supplement: S2 Fig — (TIF) [file pone.0330447.s004.tif]

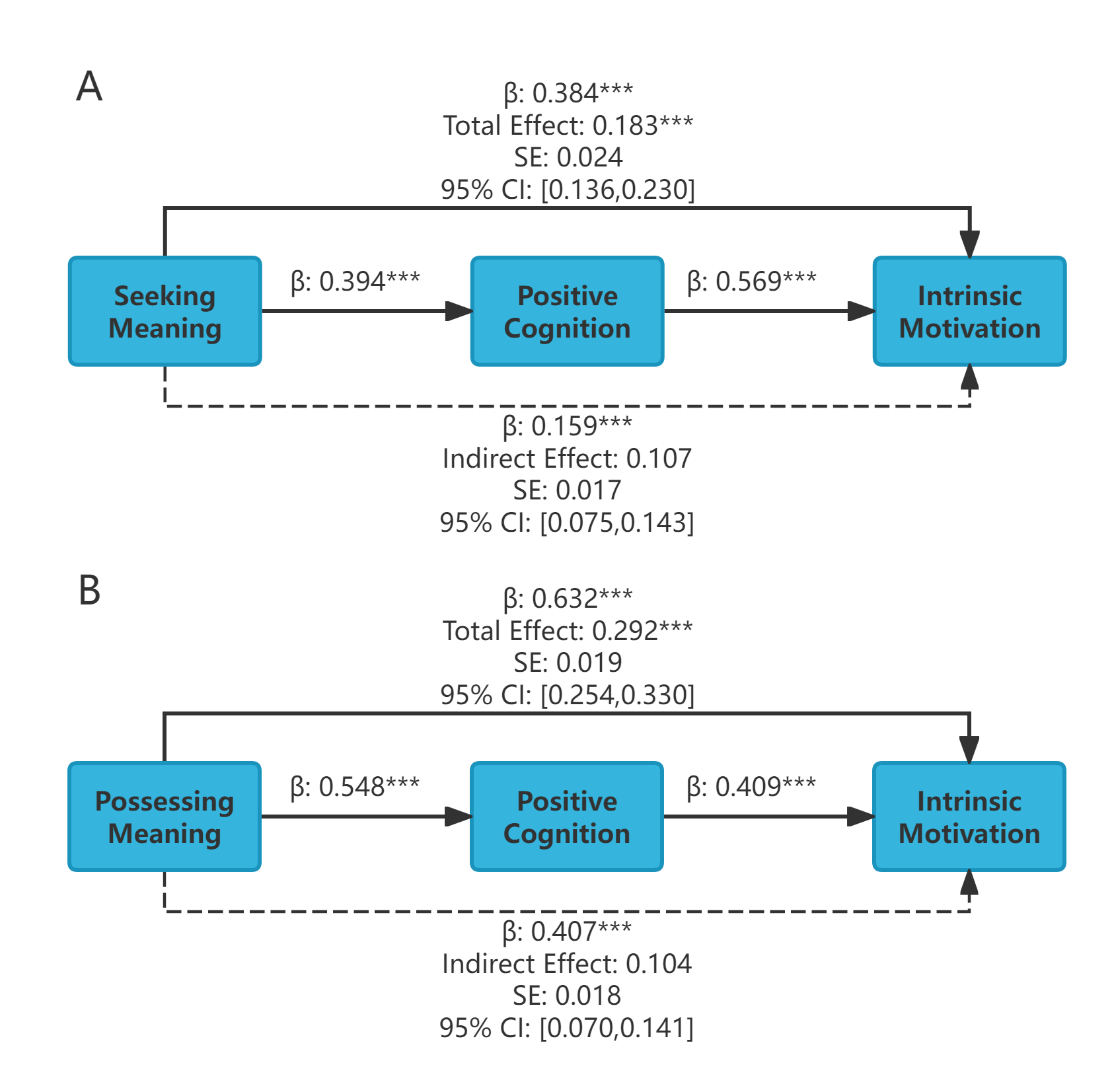

Supplement: S3 Fig — (TIF) [file pone.0330447.s005.tif]

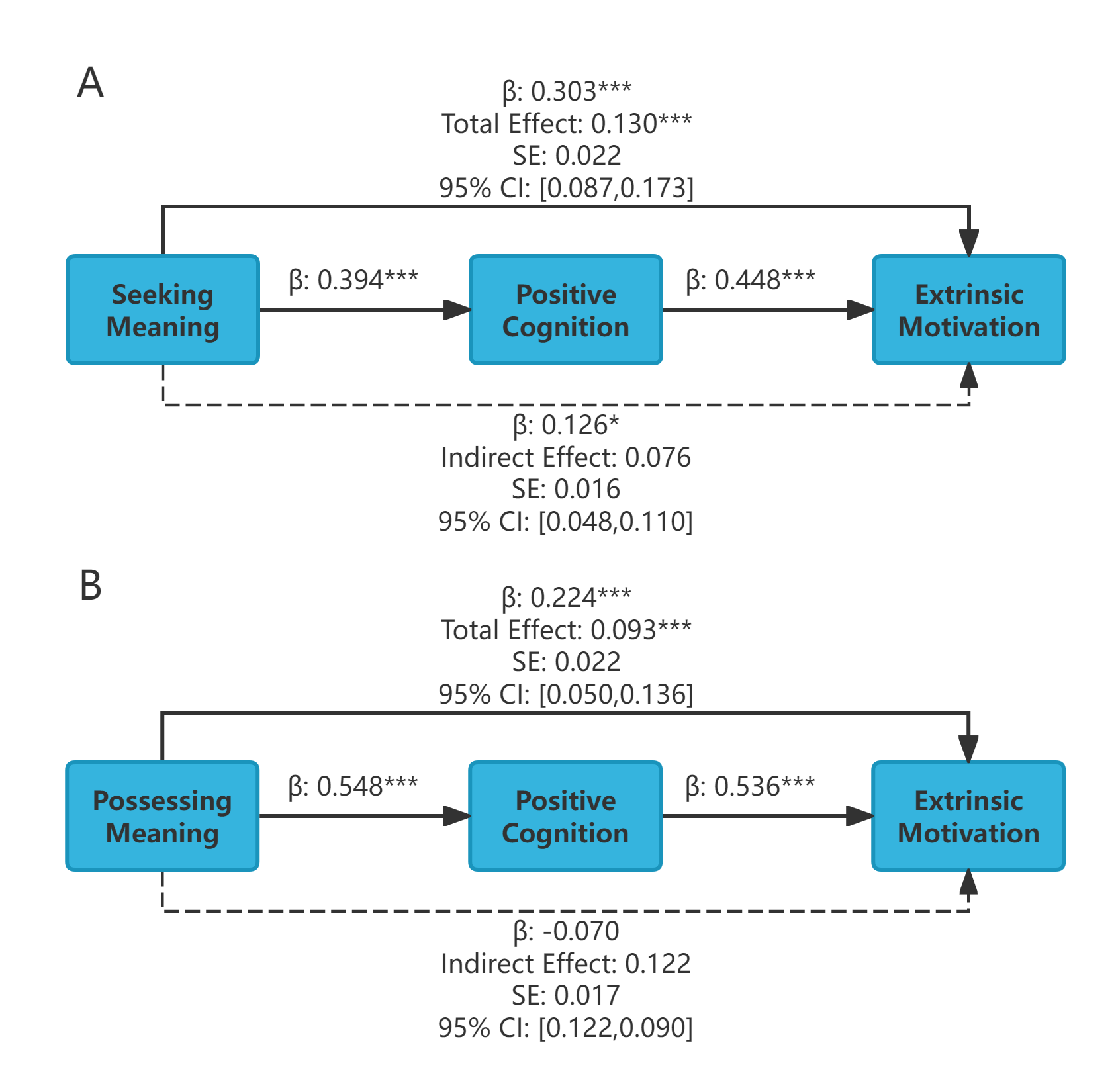

Supplement: S4 Fig — (TIF) [file pone.0330447.s006.tif]
