# Supplementary material for: Meaning in life, positive cognition, and learning motivation: A mediational analysis among Chinese college students
Source: PLoS One. 2025 Sep 12;20(9):e0330447. doi: 10.1371/journal.pone.0330447 (PMC12431252; doi:10.1371/journal.pone.0330447)

PLOS ONE

The Mediating Role of Positive Cognition in Connecting Life Meaning and Learning Motivation: A Cross-Sectional Study of Chinese College Students

--Manuscript Draft--

**Manuscript Number:**	PONE-D-25-25397
**Article Type:**	Research Article
**Full Title:**	The Mediating Role of Positive Cognition in Connecting Life Meaning and Learning Motivation: A Cross-Sectional Study of Chinese College Students
**Short Title:**	The Mediating Role of Positive Cognition in Connecting Life Meaning and Learning Motivation
**Corresponding Author:**	Zhi-ru Zhu<br>Army Medical University<br>Chongqing, CHINA
**Keywords:**	Keywords: Meaning in Life; Positive Cognition; learning motivation
**Abstract:**	Abstract<br>Background: Although existing research emphasises the direct link between life meaning and learning motivation, the psychological mechanisms linking these concepts, particularly the potential mediating role of positive cognition, have not been fully explored.<br>Objectives: This study investigated the mediating role of positive cognition in the relationship between life meaning (seeking and possessing) and learning motivation (intrinsic and extrinsic) among university students.<br>Methods: A cross-sectional survey was conducted. Data were collected from 345 university students (42.0% female) in Chongqing, China, using three validated psychological scales: the Meaning in Life Questionnaire (MLQ), Positive Cognition Questionnaire (PCQ), and Learning Motivation Questionnaire (LMQ). Hierarchical regression and bootstrapping analyses were conducted to test the mediation effects.<br>Results: Significant positive correlations were observed between life meaning, positive cognition, and learning motivation. Both dimensions of life meaning positively predicted learning motivation and positive cognition, whereas positive cognition positively predicted learning motivation. Mediation analyses indicated that positive cognition partially mediated the relationship between seeking meaning and intrinsic motivation (accounting for 58.5% of the total effect) and between possessing meaning and intrinsic motivation (35.6%). Regarding extrinsic motivation, positive cognition fully mediated the effect of possessing meaning and partially mediated the effect of seeking meaning (58.5%).<br>Conclusion: Positive cognition acts as a significant mediator in the pathway from meaning in life to learning motivation pathway. Enhancing an individual’s sense of meaning appears to foster positive cognitive habits, which in turn stimulates intrinsic motivation and amplifies extrinsic motivation. These findings highlight the potential benefits of integrating life-meaning education and positive cognitive training in academic settings to improve students’ learning engagement and overall well-being.
**Order of Authors:**	Shihong Liu
	Xiaoli Gong
	Qi Ji
	Qiaoqiao Li
	Minghui Kong
	Xiaohui Du
	Leiying Xiang
	Zhi-ru Zhu
**Additional Information:**	
**Question**	**Response**

Powered by Editorial Manager® and ProduXion Manager® from Aries Systems Corporation

**Financial Disclosure**<br><br>Enter a financial disclosure statement that describes the sources of funding for the work included in this submission. Review the submission guidelines for detailed requirements. View published research articles from *PLOS ONE* for specific examples.<br><br>This statement is required for submission and **will appear in the published article** if the submission is accepted. Please make sure it is accurate.<br><br>**Funded studies**<br>Enter a statement with the following details:<br>• Initials of the authors who received each award<br>• Grant numbers awarded to each author<br>• The full name of each funder<br>• URL of each funder website<br>• Did the sponsors or funders play any role in the study design, data collection and analysis, decision to publish, or preparation of the manuscript?<br><br>Did you receive funding for this work?	The author(s) received no specific funding for this work.
**Competing Interests**<br><br>Use the instructions below to enter a competing interest statement for this submission. On behalf of all authors, disclose any competing interests that could be perceived to bias this work—acknowledging all financial support and any other relevant financial or non-financial competing interests.<br><br>This statement is required for submission and **will appear in the published article** if the submission is accepted. Please make sure it is accurate and that any funding sources listed in your Funding Information later in the submission form are also declared in your Financial Disclosure statement.	NO authors have competing interests

View published research articles from [*PLOS ONE*]() for specific examples.<br><br>**NO authors have competing interests**<br><br>Enter: *The authors have declared that no competing interests exist.*<br><br>**Authors with competing interests**<br><br>Enter competing interest details beginning with this statement:<br><br>*I have read the journal's policy and the authors of this manuscript have the following competing interests: [insert competing interests here]*<br><br>* typeset	
**Ethics Statement**<br><br>Enter an ethics statement for this submission. This statement is required if the study involved:<br><br>• Human participants<br>• Human specimens or tissue<br>• Vertebrate animals or cephalopods<br>• Vertebrate embryos or tissues<br>• Field research<br><br>Write "N/A" if the submission does not require an ethics statement.<br><br>General guidance is provided below. Consult the submission guidelines for detailed instructions. **Make sure that all information entered here is included in the Methods section of the manuscript.**	The study was approved by the Ethics Committee of the Second Affiliated Hospital of Army Medical University (Approval No. 2023-Yan-127-01, Date: 28 September 2023). Electronic informed consent was obtained from all participants.Prior to starting the online survey, participants were presented with an introductory screen that provided essential information about the study, including its general purpose, the voluntary nature of participation, assurance of strict confidentiality and anonymity of data, and the right to withdraw from the study at any time without penalty.This introductory screen explicitly stated, "If you agree to the above, and have understood the content to be surveyed, please begin".Participants indicated their informed consent by voluntarily proceeding to complete the online questionnaire after acknowledging this statement. This electronic consent procedure was approved by the Ethics Committee. Furthermore, the online questionnaire design included an explicit option for participants to exit the survey at any point before final submission, ensuring their right to withdraw. The survey was conducted anonymously, and data were collected and analyzed in a manner that ensured participants' privacy and confidentiality.All participants were over 18 years of age.

**Format for specific study types**<br><br>**Human Subject Research (involving human participants and/or tissue)**<br>• Give the name of the institutional review board or ethics committee that approved the study<br>• Include the approval number and/or a statement indicating approval of this research<br>• Indicate the form of consent obtained (written/oral) or the reason that consent was not obtained (e.g. the data were analyzed anonymously)<br><br>**Animal Research (involving vertebrate animals, embryos or tissues)**<br>• Provide the name of the Institutional Animal Care and Use Committee (IACUC) or other relevant ethics board that reviewed the study protocol, and indicate whether they approved this research or granted a formal waiver of ethical approval<br>• Include an approval number if one was obtained<br>• If the study involved *non-human primates*, add *additional details* about animal welfare and steps taken to ameliorate suffering<br>• If anesthesia, euthanasia, or any kind of animal sacrifice is part of the study, include briefly which substances and/or methods were applied<br><br>**Field Research**<br><br>Include the following details if this study involves the collection of plant, animal, or other materials from a natural setting:<br>• Field permit number<br>• Name of the institution or relevant body that granted permission	
**Data Availability**<br><br>Authors are required to make all data underlying the findings described fully available, without restriction, and from the time of publication. PLOS allows rare exceptions to address legal and ethical concerns. See the PLOS Data Policy and FAQ for detailed information.	Yes - all data are fully available without restriction

A Data Availability Statement describing where the data can be found is required at submission. Your answers to this question constitute the Data Availability Statement and **will be published in the article**, if accepted.<br><br>**Important:** Stating 'data available on request from the author' is not sufficient. If your data are only available upon request, select 'No' for the first question and explain your exceptional situation in the text box.<br><br>Do the authors confirm that all data underlying the findings described in their manuscript are fully available without restriction?	
**Describe where the data may be found in full sentences. If you are copying our sample text, replace any instances of XXX with the appropriate details.**<br><br>• If the data are **held or will be held in a public repository**, include URLs, accession numbers or DOIs. If this information will only be available after acceptance, indicate this by ticking the box below. For example: *All XXX files are available from the XXX database (accession number(s) XXX, XXX.)*.<br>• If the data are all contained **within the manuscript and/or Supporting Information files**, enter the following: *All relevant data are within the manuscript and its Supporting Information files.*<br>• If neither of these applies but you are able to provide **details of access elsewhere**, with or without limitations, please do so. For example:<br><br>*Data cannot be shared publicly because of [XXX]. Data are available from the XXX Institutional Data Access / Ethics Committee (contact via XXX) for researchers who meet the criteria for access to confidential data.*<br><br>*The data underlying the results presented in the study are available from (include the name of the third party*	Informed consent was obtained from all participants prior to their participation. Research data supporting this publication are available at SoJump (https://www.wjx.cn/report/303121414.aspx).

and contact information or URL).<br>• This text is appropriate if the data are owned by a third party and authors do not have permission to share the data.<br><br>* typeset	
Additional data availability information:	

Manuscript

Click here to access/download;Manuscript;5-23full article-plos one.docx

The Mediating Role of Positive Cognition in Connecting Life Meaning and Learning Motivation: A Cross-Sectional Study of Chinese College Students

Shihong Liu[a1], Xiaoli Gong[b1], Qi Ji[a2], Li Qiaoqiao[a2], Minghui Kong[ac3], Xiaohui Du[a4], Leiying Xiang[ac4], Zhiru Zhu [a*]

Shihong Liu and Xiaoli Gong contributed equally to this work and share first authorship.

[a]Department of Medical Psychology, Army Medical University, Chongqing, China

[b]High Altitude Medicine Department of Army 953 Hospital, Xizang, China

[c]School of Educational Science, Chongqing Normal University, Chongqing, China

Author Emails

Shihong Liu: 19104445399@tmmu.edu.cn

Xiaoli Gong: xiaoligong0301@163.com

Qi Ji: 2753482466@qq.com

Qiaoqiao Li: liqiaoqiao@tmmu.edu.cn

Minghui Kong: kongminghui2018@163.com

Xiaohui Du: 464534148@qq.com

Leiying Xiang: 1416361857@qq.com

Zhiru Zhu: zhuzr2013@163.com

Corresponding author

*Zhiru Zhu, Army Medical University, No.30, Gaotanyan Street, Shapingba District, Chongqing 400030, China

Email: zhuzr2013@163.com

Abstract

Background: Although existing research emphasises the direct link between life meaning and learning motivation, the psychological mechanisms linking these concepts, particularly the potential mediating role of positive cognition, have not been fully explored.

Objectives: This study investigated the mediating role of positive cognition in the relationship between life meaning (seeking and possessing) and learning motivation (intrinsic and extrinsic) among university students.

Methods: A cross-sectional survey was conducted. Data were collected from 345 university students (42.0% female) in Chongqing, China, using three validated psychological scales: the Meaning in Life Questionnaire (MLQ), Positive Cognition Questionnaire (PCQ), and Learning Motivation Questionnaire (LMQ). Hierarchical regression and bootstrapping analyses were conducted to test the mediation effects.

Results: Significant positive correlations were observed between life meaning, positive cognition, and learning motivation. Both dimensions of life meaning positively predicted learning motivation and positive cognition, whereas positive cognition positively predicted learning motivation. Mediation analyses indicated that positive cognition partially mediated the relationship between seeking meaning and intrinsic motivation (accounting for 58.5% of the total effect) and between possessing meaning and intrinsic motivation (35.6%). Regarding extrinsic motivation, positive cognition fully mediated the effect of possessing meaning and partially mediated the effect of seeking meaning (58.5%).

Conclusion: Positive cognition acts as a significant mediator in the pathway from meaning in life to learning motivation pathway. Enhancing an individual’s sense of meaning

appears to foster positive cognitive habits, which in turn stimulates intrinsic motivation and amplifies extrinsic motivation. These findings highlight the potential benefits of integrating life-meaning education and positive cognitive training in academic settings to improve students' learning engagement and overall well-being.

Keywords: Meaning in Life; Positive Cognition; Learning Motivation

Glossary

1.Meaning in Life

Definition: An individual's perception of the purpose, significance, and coherence of their life.

Dimensions distinguished in this study

Search for Meaning (or Seeking Meaning): The active pursuit or desire to find meaning and purpose in life; a state or behaviour characterised by this search.

Presence of Meaning (or Possessing Meaning): The degree to which an individual subjectively perceives their current life as having meaning and purpose.

2.Learning Motivation:

Definition: The internal drive or desire that directs student behaviour towards learning goals.

Types distinguished in this study

Intrinsic Motivation: Motivation stemming from internal factors, such as interest, enjoyment, or satisfaction derived from the learning activity itself.

Extrinsic Motivation: Motivation driven by external rewards or pressures, such as grades, praise, avoidance of punishment, or future career benefits.

3.Positive Cognition:

Definition: Refers to an individual's tendency or cognitive habit of interpreting experiences, evaluating the self, and anticipating the future in an optimistic, positive, and constructive manner.

4.Mediation (or Mediating Role):

Definition: Refers to the mechanism or process through which one variable (the mediator, e.g. positive cognition in this study) accounts for the relationship between another variable (the independent variable, e.g. meaning in life) and a third variable (the dependent variable, e.g. learning motivation). This explains how or why the independent variable influences the dependent variable.

5.Cross-sectional Survey:

Definition: A research method involving the collection of data from a sample population at a single point in time to examine the relationships between variables.

6.Hierarchical Regression Analysis

Definition: A statistical technique is used to assess the relationship between variables by entering predictor variables into a regression model in predetermined steps or blocks (hierarchies), allowing for the evaluation of the unique contribution of variables while controlling for others.

7.Bootstrapping Analysis:

Definition: A resampling statistical method. In this study, it is primarily employed to test the statistical significance of indirect effects and is particularly useful for constructing confidence intervals for these effects, which are often considered more robust than traditional methods under non-normality.

1.Introduction

Amidst the rapid national development and economic advancement in China, there has been a notable increase in material standards of living. However, commensurate improvements in subjective well-being have not been uniformly observed, and complex trends, including potential stagnation or decline, have been noted within certain population segments. Intensified social competition and rapid societal transformations may contribute to existential questioning among some individuals, particularly university students (1) . Historically, while emphasising academic achievement, the educational system in China may have devoted insufficient attention to the cultivation of students' sense of meaning in life (MiL) and their holistic psychological development. Recent occurrences of incidents related to student mental health underscore the imperative of fostering healthy perspectives on life and values, alongside promoting positive self-perception, during formative education (2) .

Meaning in Life (MiL) is generally conceptualised as an individual's subjective perception of the purpose, significance, and coherence of their existence (3, 4) . It is considered a critical component of psychological well-being and positive adaptation. Early existential philosophers addressed the individual construction of meaning in life. Contemporary psychological research, particularly within positive psychology, identifies 'life goals' or 'purpose' as a core constituent of MiL, providing substantive content to the sense of meaning (5) . Researchers commonly distinguish between two dimensions of MiL: the presence of meaning, reflecting the degree to which individuals currently perceive their lives as meaningful, and the search for meaning, denoting the active process and motivation to find or establish life meaning (6) . This distinction remains salient in the current research, as the presence of meaning

is typically associated with enhanced well-being and adjustment, whereas the implications of the search for meaning appear more complex (2, 7).

A substantial body of recent research confirms significant positive correlations between MiL (or its core components, such as a sense of purpose) and university students' learning engagement, academic motivation, and academic achievement (8, 9) . Possessing a clear sense of life can provide an intrinsic drive for students' learning; understanding the value and objectives of their studies helps stimulate academic enthusiasm (7) . Consequently, integrating meaning-in-life ed ucation into pedagogical practices and guiding students to explore personal values and life goals may not only contribute to the development of their life perspectives, but also positively influence their learning motivation (10). Learning motivation, the internal force propelling student learning behaviour, encompasses intrinsic motivation, directed towards the inherent enjoyment and satisfaction of the learning activity itself, and extrinsic motivation, driven by external rewards, punishments, or pressures (e.g. grades, peer evaluation) (11, 12) . In the contemporary context, advocating for lifelong and autonomous learning and stimulating and sustaining university students' learning motivation, particularly intrinsic motivation, is critically important (7, 13) .

Although the direct association between MiL and learning motivation has received considerable empirical support (2, 8) , the underlying psychological mechanisms warrant further investigation. Positive Cognition—defined herein as an individual's habitual tendency to interpret experiences, evaluate the self, and anticipate the future in an optimistic, positive, and constructive manner—is proposed as a potential mediating factor. The experience of MiL inherently involves cognitive appraisal processes; individuals who perceive life as meaningful

typically exhibit more positive cognitive patterns (14) . Recent studies have highlighted the role of positive psychological resources (such as psychological capital, encompassing optimism, hope, and self-efficacy) in fostering academic engagement and well-being (15, 16) , concepts closely related to positive cognition. Individuals with positive cognitions may more readily discover or construct meaning in life, thereby deriving growth(15) This positive cognitive style— characterised by focusing on positive information, maintaining hope for the future, and believing in one's capacity to cope with challenges—is crucial for sustaining learning effort, setting, and pursuing academic goals, and consequently may influence learning motivation (17) . Previous research has also provided preliminary evidence suggesting links between positive cognition or related constructs (e.g. attention to positive information and perspective-taking) and MiL experience (18, 19) .

Drawing upon the aforementioned theoretical background and empirical findings, this study hypothesises that positive cognition mediates the relationship between meaning in life and learning motivation. Specifically, the present study aimed to investigate: 1) whether meaning in life (presence and search) and positive cognition positively predict learning motivation (intrinsic and extrinsic), and 2) whether positive cognition functions as a mediator in the association between meaning in life and learning motivation. Elucidating this mediating mechanism will contribute to a deeper understanding of how meaning in life influences students' academic drive and provide targeted empirical evidence for developing interventions aimed at enhancing university students' learning motivation and promoting their psychological health.

2. Method

2.1 Participants

A total of 351 college students from a university in Chongqing were selected using random sampling. The recruitment period for this study was from 01/03/2024 to 01/08/2024. All participants were over 18 years of age.After removing missing data and careless responses from the dataset, the final analysis included 345 valid participants, resulting in an efficacy rate of 98.29%. The mean age of subjects was 21.01 ± 2.52 years, with 200 males (21.29 ± 2.47 years) and 145 females (20.64 ± 2.54 years).

The study was approved by the Ethics Committee of the Second Affiliated Hospital of Army Medical University (Approval No. 2023-Yan-127-01, Date: 28 September 2023). Electronic informed consent was obtained from all participants.Prior to starting the online survey, participants were presented with an introductory screen that provided essential information about the study, including its general purpose, the voluntary nature of participation, assurance of strict confidentiality and anonymity of data, and the right to withdraw from the study at any time without penalty.This introductory screen explicitly stated, "If you agree to the above, and have understood the content to be surveyed, please begin".Participants indicated their informed consent by voluntarily proceeding to complete the online questionnaire after acknowledging this statement. This electronic consent procedure was approved by the Ethics Committee. Furthermore, the online questionnaire design included an explicit option for participants to exit the survey at any point before final submission, ensuring their right to withdraw. The survey was conducted anonymously, and data were collected and analyzed in a manner that ensured participants' privacy and confidentiality.

2.2 ~~Tools~~

2.2.1 Meaning in Life Questionnaire (MLQ)

The questionnaire, originally developed by Steger in 2006 and revised by Liu Sisi in 2010, includes two aspects: the Presence of Meaning (MLQ-P) and Search for Meaning (MLQ-S) (20) . It includes nine items, with items 6, 7, 8, and 9 measuring the Presence of Meaning dimension and items 1, 2, 3,4, and 5 measuring the Search for Meaning dimension. The second item was reverse-scored. The questionnaire was based on a Likert 7-point scale, ranging from "Completely Disagree" (1 point) to "Completely Agree" (7 points). The higher the score, the stronger the meaning of life. In this study, the Cronbach's α coefficient was 0.855 for the entire questionnaire, 0.892 for the Presence of Meaning subscale, and 0.849 for the Search for Meaning subscale.

2.2.2 Positive Cognitive Questionnaire

The Chinese version of the Positive and Negative Information Attention Scale, revised by Feng et al., was adopted. This study used the Positive Information Attention subscale (21) . The subscale comprises 12 items rated on a Likert 5-point scale: "5 (Completely Agree)," "4 (Mostly Agree)," "3 (Uncertain)," "2 (Mostly Disagree)," and "1 (Completely Disagree)." Higher scores reflect a stronger positive cognitive bias. The internal consistency (Cronbach's α) for the positive cognitive subscale was 0.928.

2.2.3 Learning Motivation Questionnaire

Learning motivation is generally categorised into two dimensions: intrinsic and

extrinsic. This study used the two-dimensional learning motivation scale designed by Wei, which consists of 16 items divided into intrinsic and extrinsic motivation scales (Cronbach's α coefficients: 0.883 and 0.710) (22) .

2.3 Statistical Analysis

SPSS 24.0 was used for data preprocessing, descriptive analysis, and correlation analysis. The mediating role of positive cognition was investigated by applying the hierarchical regression analysis proposed by Wen (23) . The significance of the mediation effect was verified using the bootstrap method.

3 Results

3.1 Common Method Bias

Harman's single-factor test was used to assess common method bias. Exploratory factor analysis revealed six factors with eigenvalues greater than one, accounting for a total variance of 62.79%. The first factor accounted for 35.87% of the variance, which was below the critical threshold of 40%, indicating no significant common method bias.

3.2 Reliability and Validity Tests

3.2.1 Meaning in Life Questionnaire

The fit indices for the MIL Questionnaire were $\chi^2/df = 2.98$, RMSEA = 0.08, CFI = 0.97, NNFI = 0.96, and RMR = 0.12. The Pearson correlation coefficients between other factors were smaller than the square root of the average variance extracted (AVE) values. For the search for meaning subscale, the AVE was 0.59 (> 0.50) and Composite Reliability (CR) was

0.85 (> 0.70), indicating strong convergent validity. For the meaning subscale, the AVE was 0.64 (> 0.50) and CR was 0.90 (> 0.70), also demonstrating good convergent validity.

3.2.2 Learning Motivation Scale

The first-order confirmatory model fit indices for the Learning Motivation Scale were $\chi^2/df = 5.22$, RMSEA = 0.11, CFI = 0.81, NNFI = 0.78, and RMR = 0.09. The Pearson correlation coefficients between the other factors were smaller than the square root of the AVE values. For intrinsic motivation, the AVE was 0.51 (> 0.50) and CR was 0.89 (> 0.70), indicating strong convergent validity. For extrinsic motivation, the AVE was 0.28, and CR was 0.72 (> 0.70), indicating acceptable convergent validity.

3.2.3 Positive Cognition Scale

The first-order confirmatory model fit indices for the Positive Cognition Scale were $\chi^2/df = 6.52$, RMSEA = 0.13, CFI = 0.89, NNFI = 0.86, and RMR = 0.04. The Pearson correlation coefficients between the other factors were smaller than the square root of the AVE values. The AVE for positive cognition was 0.53 (> 0.50) and CR was 0.93 (> 0.70), indicating strong convergent validity.

Discriminant Validity: The absolute value of the correlation between potential variables was generally less than the square root of their respective AVE values (Table 1), confirming discriminant validity.

3.3 Descriptive Statistics and Correlation Analysis

Descriptive statistics and correlations between meaning in life, learning motivation,

and positive cognition are presented in Table 2. The results showed significant positive correlations between life meaning and learning motivation, life meaning and positive cognition, and positive cognition and learning motivation.

3.4 Mediation Analysis of Positive Cognition

3.4.1 Mediation of Positive Cognition Between Meaning in Life and Intrinsic Motivation

The hierarchical regression analysis method proposed by Wen Zhonglin was used to examine the mediating role of positive cognition between a sense of meaning in life and intrinsic motivation. First, we test Equation (1), where $y = cx + e1$. If c was not significant, the intermediate effect test was stopped. If the c-value is significant, Equation 2 is used. After passing the significance test, Equation $M = ax + e2$ was used. If a was not significant, the testing was stopped. If a is significant, then the next equation is tested. Finally, after both Equations 1 and 2 pass the significance tests, Equation 3 can be tested, that is, $y = c'x + bM + e3$, to test the significance of b. If b indicates significance, it indicates a significant mediating effect. When testing c, if c is not significant, it indicates a complete mediating effect; if it is significant, it suggests a partial mediating effect, meaning that M can influence the effect of x on y.

Hierarchical regression analysis and the bootstrap method were employed to test the establishment of a mediating effect using SPSS 24.0. The results of the stepwise regression analysis are presented in Table 3, and the bootstrap mediating effect results are presented in Table 4.

From Table 3, it can be seen that in Step 1, the standardized regression coefficient for the independent variable seeking meaning was 0.384, $t = 7.693$, $p < 0.001$, and for the

independent variable possessing meaning, it was 0.632, $t = 15.085$ $p < 0.001$, indicating that both seeking meaning and possessing meaning positively influence intrinsic motivation, validating coefficient c; in Step 2, the standardized regression coefficient for the independent variable seeking meaning was 0.394, $t = 7.946$, $p < 0.001$, and for the independent variable possessing meaning, it was 0.548, $t = 12.139$, $p < 0.001$, indicating that both seeking meaning and possessing meaning positively influence positive cognition, validating coefficient a; in Step 3, when the independent variable is seeking meaning, the standardized regression coefficient for the mediating variable positive cognition was 0.569, $t = 12.721$, $p < 0.01$, when the independent variable is possessing meaning, the standardized regression coefficient for the mediating variable positive cognition was 0.409, $t = 9.089$, $p < 0.01$, indicating that positive cognition positively influences intrinsic motivation, validating coefficient b, and showing that positive cognition plays a mediating role between the sense of meaning in life and intrinsic motivation. Meanwhile, the standardised regression coefficient for the independent variable seeking meaning was 0.159, $t = 3.553$, $p < 0.001$, and for the independent variable with meaning, it was 0.407, $t = 9.055$, $p < 0.001$, validating the coefficient c’, indicating that positive cognition plays a partial mediating role between the sense of meaning in life and intrinsic motivation.

Table 4 shows that the total effect value of the mediating path from seeking meaning to positive cognition to intrinsic motivation was 0.183 with a 95% confidence interval of [0.136, 0.230], which did not include zero, indicating that the total effect was valid. The indirect effect value was 0.107, with a 95% confidence interval of [0.075–0.143], which did not include 0, indicating that the mediating effect was valid. The direct effect value was 0.076, with a 95% confidence interval of [0.034–0.118], which did not include 0, indicating a partial mediating

effect. The proportion of the mediating effect to the total effect was 0.107/0.183 × 100% = 58.5%, indicating that 58.5% of the impact of seeking meaning on intrinsic motivation was through positive cognition.

The total effect value of the mediating path from possessing meaning to positive cognition to intrinsic motivation was 0.292, with a 95% confidence interval of [0.254, 0.330], which did not include zero, indicating that the total effect was valid. The indirect effect value was 0.104, with a 95% confidence interval of [0.070–0.141], which did not include zero, indicating that the mediating effect was valid. The direct effect value was 0.189, with a 95% confidence interval of [0.148, 0.229], which did not include 0, indicating a partial mediating effect. The proportion of the mediating effect to the total effect was 0.104/0.292 × 100% = 35.6%, indicating that 35.6% of the impact of possessing meaning on intrinsic motivation was through positive cognition.

Mediating effects and path coefficient diagrams were drawn based on the results of the comprehensive mediating effects, as shown in Figure 1.

3.4.2 The Mediating Effect of Positive Cognition between Sense of Life Meaning and Extrinsic Motivation

Using SPSS 24.0 for hierarchical regression analysis and the bootstrap method to test the validity of the mediating effect, the results of the stepwise regression analysis are shown in Table 5, and the bootstrap mediating effect results are shown in Table 6.

From Table 5, it can be seen that in Step 1, the standardised regression coefficient of the independent variable seeking meaning is 0.303, $t = 5.889$, $p < 0.001$, and the standardised regression coefficient of the independent variable with meaning is 0.224, $t = 4.258$, $p < 0.05$,

indicating that both seeking meaning and possessing meaning positively affect extrinsic motivation, thus verifying coefficient c. In Table 2, the standardised regression coefficient of the independent variable seeking meaning was 0.394, $t = 7.946$, $p < 0.001$, and the standardised regression coefficient of the independent variable with meaning was 0.548, $t = 12.139$, $p < 0.001$, indicating that both seeking meaning and possessing meaning positively affected positive cognition, thus verifying Coefficient a. On Tep 3, when the independent variable was seeking meaning, the standardised regression coefficient for the mediating variable, positive cognition, was 0.448 ($t = 8.860$, $p < 0.001$). When the independent variable possessed meaning, the standardised regression coefficient for the mediating variable, positive cognition, was 0.536 ($t = 9.584$, $p < 0.001$), indicating that positive cognition positively affects extrinsic motivation. This verifies coefficient b, indicating that positive cognition plays a mediating role between sense of life meaning and extrinsic motivation. The standardised regression coefficient of the independent variable seeking meaning was 0.126 ($t = 2.499$, $p < 0.05$), indicating that positive cognition partially mediated the relationship between seeking meaning and extrinsic motivation. The standardized regression coefficient of the independent variable with meaning was -0.070, $t = -1.249$, $p > 0.05$, not verifying the coefficient c', indicating that positive cognition plays a complete mediating role between possessing meaning and extrinsic motivation.

From Table 6, it can be seen that the total effect value of the mediating path from seeking meaning to positive cognition to extrinsic motivation is 0.130, with a 95% confidence interval of [0.087, 0.173], which does not include 0, indicating that the total effect is valid. The indirect effect value was 0.076, with a 95% confidence interval of [0.048, 0.110], which did not include zero, indicating that the mediating effect was valid. The direct effect value was

0.054, with a 95% confidence interval of [0.012–0.097], which did not include 0, indicating a partial mediating effect. The proportion of the mediating effect to the total effect was 0.076/0.130 × 100% = 58.5%, indicating that 58.5% of the impact of seeking meaning on extrinsic motivation occurred through positive cognition.

The total effect value of the mediating path from meaning possession to positive cognition to extrinsic motivation was 0.093, with a 95% confidence interval of [0.050, 0.136], which did not include zero, indicating that the total effect was valid. The indirect effect value was 0.122, with a 95% confidence interval of [0.090,0.159], which did not include 0, indicating that the mediating effect was valid. The direct effect value was -0.029, with a 95% confidence interval of [-0.075, 0.017], which included 0, indicating a complete mediating effect. This finding indicates that the effect of possessing meaning on extrinsic motivation occurs primarily through positive cognition.

Mediating effects and path coefficient diagrams were drawn based on the results of the comprehensive mediating effects, as shown in Figure 2.

4 Discussion

This study investigated the interrelationships between meaning in life, positive cognition, and learning motivation in a sample of Chinese university students, with a specific focus on examining the mediating role of positive cognition in the pathway from meaning in life to learning motivation. These findings contribute novel empirical evidence to the understanding of how these crucial psychological constructs interact within academic settings.

4.1 Interrelationships among Meaning in Life, Positive Cognition, and Learning

Motivation

First, the findings revealed significant positive correlations between meaning in life (encompassing both the presence of meaning and search for meaning dimensions) and learning motivation (including intrinsic and extrinsic motivation) among university students. Specifically, students reporting higher levels of meaning in life experiences, irrespective of whether they perceived their lives as currently meaningful (presence) or actively seeking meaning (search), exhibited greater learning motivation. This aligns strongly with recent international and domestic research on university student populations (2,8,9), which consistently identifies meaning in life or a sense of purpose as a key predictor of academic engagement and motivation. As Villani et al. (7) suggested, a clear sense of purpose can provide the essential 'why' for learning, thereby driving academic behaviours. The observation in our study that both dimensions of meaning in life positively predicted both types of motivation suggests that, within the Chinese cultural context, both the current appreciation of meaning and pursuit of future goals may synergistically foster university students' learning drives.

Second, the results further corroborate the strong association between meaning in life and positive cognition. Students who experienced their lives as meaningful demonstrated a greater tendency towards positive and optimistic cognitive processing. This supports Heintzelman and King's (14) perspective that the experience of meaning inherently involves positive cognitive appraisals. Recent research has increasingly highlighted the role of positive psychological resources, such as psychological capital (PsyCap), which encompasses components such as optimism and hope akin to the positive cognition construct used here, as crucial links between meaning in life, well-being, and academic adaptation (15,16). Individuals

possessing or searching for meaning may cultivate positive cognitive habits (e.g. focusing on positive aspects, constructively interpreting challenges) to sustain or enhance their sense of meaning, potentially leading to greater psychological resilience and personal growth (1,24).

Third, this study revealed a significant positive correlation between positive cognition and learning motivation, indicating that students with higher levels of positive cognition also reported greater learning motivation. This finding is consistent with theoretical expectations: individuals with a positive cognitive orientation may possess greater self-efficacy (25), demonstrate more resilience when facing academic challenges (17), and derive greater satisfaction from the learning process, thereby fostering both intrinsic interest and motivation to pursue extrinsic outcomes. Such students may be more inclined to set and persevere towards academic goals, showing less propensity to disengage when encountering difficulties, potentially establishing a virtuous cycle where positive cognition fuels motivation, leading to positive outcomes that further reinforce positive cognition.

4.2 The Mediating Role of Positive Cognition between Meaning in Life and Learning Motivation

A central finding of this study lies in illuminating the significant mediating role of positive cognition in the pathway linking meaning in life to learning motivation.

Specifically, our mediation analyses indicated that positive cognition partially mediated the relationship between both the presence of meaning and search for meaning and intrinsic motivation. Based on our results, the presence of meaning and the search for meaning account for 58.5% and 35.6% of the total effect, respectively, and a significant portion of the total effect of meaning in life (both presence and search) on intrinsic motivation was channelled

through positive cognition. This suggests that while meaning in life can directly foster students' inherent interest and enjoyment in learning (intrinsic motivation), a substantial part of its impact occurs indirectly by fostering positive cognitive styles. Experiencing or searching for meaning seems to cultivate optimism and perhaps self-efficacy, thereby igniting intrinsic academic passion. This finding resonates with Self-Determination Theory (11) , which emphasises the roles of competence and autonomy – potentially bolstered by positive cognition – in driving intrinsic motivation.

Regarding extrinsic motivation, the findings revealed distinct mediation patterns. Positive cognition was found to partially mediate the relationship between search for meaning and extrinsic motivation, accounting for 58.5% of the total effect, while it fully mediated the link between presence of meaning and extrinsic motivation. This implies that the impetus derived from the current experience of life as meaningful (presence of meaning) towards pursuing external academic goals (e.g. achieving good grades, future career prospects) operates almost entirely through positive cognitive appraisals, such as optimistic expectations of success. Conversely, the active process of searching for meaning appears to influence extrinsic motivation both directly (perhaps viewing external achievements as instrumental to finding meaning) and indirectly (fostering positive cognition). These differentiated mediation pathways highlight positive cognition as a crucial psychological mechanism that translates the broader experience of meaning in life into motivation for specific academic behaviours, especially concerning the pursuit of external objectives. This observation is consistent with recent research trends emphasising the mediating/moderating roles of psychological resources (e.g. psychological capital, social support) in linking well-being indicators (e.g. life satisfaction,

meaning) to academic outcomes such as engagement and burnout (2, 9, 15,) .

In sum, these findings suggest that while fostering meaning in life among university students is crucial, interventions might be more effective in stimulating and sustaining learning motivation if they concurrently cultivate positive cognitive habits. From the perspective of need theories (e.g. Alderfer's ERG theory and Maslow's hierarchy), intrinsic motivation is often associated with growth needs (closely related to meaning), whereas extrinsic motivation tends to align with existence, relatedness, or esteem needs. Our results imply that positive cognition may function as a critical psychological 'converter', enabling individuals to translate their deep-seated sense of life meaning into the confidence and drive necessary for pursuing goals across various need levels, especially those externally oriented.

5.Conclusions

(1) Significant positive correlations were found between meaning in life (both presence and search dimensions), positive cognition, and learning motivation (both intrinsic and extrinsic) in a sample of Chinese university students. Meaning in life positively predicted both learning motivation and positive cognition. Furthermore, positive cognition positively predicted learning motivation.

(2) Positive cognition played a significant mediating role in the relationship between meaning in life and learning motivation. Specifically:

* Positive cognition partially mediated the relationship between both the presence of meaning and search for meaning and intrinsic motivation.

* Positive cognition partially mediates the relationship between search for meaning and

extrinsic motivation.

* Positive cognition fully mediates the relationship between the presence of meaning and extrinsic motivation.

These findings indicate that enhancing university students' meaning in life, potentially achieved through improving their levels of positive cognition and cultivating adaptive cognitive habits, may serve to partially stimulate intrinsic learning motivation while substantially (or, in the case of the presence of meaning, fully) strengthening extrinsic learning motivation.

6.Limitations and Future Directions

First, the adoption of a cross-sectional design precludes the determination of dynamic developmental trajectories and causal relationships between meaning in life, positive cognition, and learning motivation. Future investigations could employ longitudinal designs or cross-lagged panel models, particularly when integrated with meaning-centred educational programs or positive psychological interventions (1) , to achieve a more profound understanding of the temporal dynamics and reciprocal influences of these variables. This would provide a more robust evidence base for the formulation of effective intervention strategies.

Second, the study sample was predominantly recruited from a single university in the Chongqing municipality, potentially limiting the generalisability of the findings. Future research should endeavour to recruit more diverse samples encompassing various geographical regions, types of higher education institutions, and academic disciplines to enhance the external validity of the results. Concurrently, extending the research population to other developmental cohorts, such as adolescents and early career adults, would facilitate a broader understanding of the developmental trajectory and functional role of these psychological constructs across the

lifespan.

Furthermore, this study primarily focused on positive cognition as the sole mediating variable. Subsequent research could benefit from exploring additional potential mediators or moderators, including self-efficacy, psychological resilience, social support (1, 2) , coping strategies, and specific cultural values. Investigating these factors would enable the development of more integrated and comprehensive theoretical models that elucidate the complex mechanisms through which meaning in life affects learning motivation.

7. Declarations

This study was supported by grants from the Foundation of Basic Research and the Teaching Reform Project of the Army Medical University (Nos. Y2025W14 & 2022B12). All procedures involving human participants were conducted in accordance with the ethical standards of the Institutional Review Board (IRB) of the Second Affiliated Hospital of Army Medical University (Approval No. 2023-Yan-127-01, Date: 28 September 2023). Informed consent was obtained from all participants prior to their participation. Research data supporting this publication are available at SoJump (https://www.wjx.cn/report/303121414.aspx).The authors declare that they have no competing financial or personal relationships that could have influenced the work reported in this study. Generative AI tools have only been used to improve language clarity and readability. The authors take full responsibility for the content and integrity of this manuscript. All authors have approved the manuscript and agree with its submission to the journal. The corresponding author, Zhi-Ru Zhu, takes full responsibility for communicating with the journal during the review.

8. Author Contributions

All authors contributed substantially to the work reported in this manuscript. Shihong Liu contributed to the conceptualization, methodology, and writing of the original draft. Xiaoli Gong was responsible for data curation, validation, and visualization. Minghui Kongand Qi Ji conducted formal analyses. Qiaoqiao Li and Qi Ji conducted experiments. Leiying Xiang and Xiaohui Du supervised the study. Zhiru Zhu secured funding, managed the project administration, provided resources, and supervised the review and editing of the manuscript. Shihong Liu and Xiaoli Gong contributed equally to this work and share first authorship. All authors have read and approved the final version of the manuscript and agreed to be accountable for all aspects of the study.

Reference

1. Wu C, Liu X, Liu J, Tao Y, Li Y. Strengthening the meaning in life among college students: the role of self-acceptance and social support - evidence from a network analysis. Front Psychol. 2024;15. http://doi.org/10.3389/fpsyg.2024.1433609
2. Chen C, Zhu Y, Xiao F, Que M. Academic motivation and social support: mediating and moderating the life satisfaction and learning burnout link. Psychol Res Behav Ma. 2023:4583-98.
3. Martela F, Steger MF. The three meanings of meaning in life: Distinguishing coherence, purpose, and significance. J Posit Psychol. 2016;11(5):531-45. http://doi.org/10.1080/17439760.2015.1137623
4. Michael F. Stegera SOTB. The Journal of Positive Psychology: Dedicated to furthering research and promoting good practice. The Journal of Positive Psychology. 2009;4(1):43-52. http://doi.org/10.1080/17439760802303127
5. Emmons RA. Personal goals, life meaning, and virtue: Wellsprings of a positive life.. 2003.
6. Steger MF, Frazier P, Oishi S, Kaler M. The meaning in life questionnaire: Assessing the presence of and search for meaning in life. J Couns Psychol. 2006;53(1):80-93. http://doi.org/10.1037/0022-0167.53.1.80
7. Yuliawati L, Virlia S, Sandjaja M, Sari TP, Sihombing B, Dorkas MA, et al. Could Purpose in Life Help Unmotivated Students to Thrive? When Motivation and Purpose in Life Matter for Peer Mentoring. Psychol Res Behav Ma. 2023;16:3139-49. http://doi.org/10.2147/PRBM.S425161
8. Ahmad LKRA. A Comparative Study Of The Meaning Of Life, Academic Motivation, Learning Styles, Study Habits, And Academic Achievement Between University And College Students In Different Educational Institutions. Migr Lett. 2024;21(S14). http://doi.org/doi.org/10.59670/ml.v21iS14.11300
9. Chukwuorji JC, Agbo A, Ajaero CK, Ifeagwazi CM. Presence of meaning in life and subjective well-being as mediators of association between sense of community and academic engagement. Revista Colombiana De Psicología. 2024;33(2):85-102.
10. Steger MF. Experiencing meaning in life: Optimal functioning at the nexus of well-being, psychopathology, and spirituality.: Routledge; 2013. p. 165-84.
11. Ryan RM, Deci EL. Intrinsic and Extrinsic Motivations: Classic Definitions and New Directions. Contemp Educ Psychol. 2000;25(1):54-67. http://doi.org/10.1006/ceps.1999.1020
12. Trevino NN, DeFreitas SC. The relationship between intrinsic motivation and academic achievement for first generation Latino college students. Soc Psychol Educ. 2014;17:293-306.
13. Badubi RM. Theories of motivation and their application in organizations: A risk analysis. International Journal of Innovation and Economic Development. 2017;3(3):44-51.
14. Heintzelman SJ, King LA. Life is pretty meaningful. Am Psychol. 2014;69(6):561.
15. Borges AG, Peñalver J, Martínez IM, Salanova M. Academic engagement in university students. The mediator role of Psychological Capital as personal resource. Educ XX1. 2023;26(2):51-70.
16. Xu J, Choi M. Can emotional intelligence increase the positive psychological capital and life satisfaction of Chinese university students? Behav Sci-Basel. 2023;13(7):614.
17. Demirtaş AS. Optimism and happiness in undergraduate students: Cognitive flexibility and adjustment to university life as mediators. Anales De Psicología/Annals of Psychology. 2020;36(2):320-9.
18. Shuyue Z, Yan X. A Diary Study of the Relationship Between Event Cognition and Meaning in

Life. Chinese Journal of Special Education. 2013;(11):5.
19. Yu W, Xinyang W, Yiqun G. Effect of search for meaning and perspective change on depression in college students: A mediated moderation model. Chinese Mental Health Journal. 2015;29(11):6.
20. Sisi L, Yiqun G. Reliability and validity of the Chinese version of the Meaning in Life Questionnaire. Chinese Mental Health Journal. 2010;24(006):478-82.
21. Qin D, Zhengzhi F, Jing L, Yunbo L, Xiuna L. Validity and reliability of the Chinese Attention to Positive and Negative Inventory in college students. Chinese Mental Health Journal. 2015;29(5):6.
22. Wei W. A Comparative Study of Self-Efficacy, Learning Motivation, and Achievement Goal Orientation Between High-Achieving and Underachieving College Students: Nanjing Normal University; 2006.
23. Zhonglin W, Lei Z, Jietai H, Hongyun L. Testing and Application of the Mediating Effects. Acta Psychologica Sinica. 2004;36(5):7.
24. Lee V, Cohen SR, Edgar L, Laizner AM, Gagnon AJ. Meaning-making intervention during breast or colorectal cancer treatment improves self-esteem, optimism, and self-efficacy. Social Science & Medicine. 2006;62(12):3133-45.
25. Bandura A. Self-efficacy: The exercise of control.: Macmillan; 1997.

Table 1 Validity results of variables

	AVE	CR	Positive Cognition	Seeking Meaning	Possessing Meaning	Intrinsic Motivation	Extrinsic Motivation
Positive Cognition	0.53	0.93	0.730				
Seeking Meaning	0.59	0.85	0.394	0.770			
Possessing Meaning	0.64	0.90	0.548	0.326	0.798		
Intrinsic Motivation	0.51	0.89	0.632	0.384	0.632	0.715	
Extrinsic Motivation	0.28	0.72	0.498	0.303	0.224	0.398	0.526

Diagonal numbers represent the square root of AVE

Table 2 Descriptive statistics and Pearson correlations analysis of variables

	M	SD	Positive Cognition	Seeking Meaning	Possessing Meaning	Intrinsic Motivation	Extrinsic Motivation
Positive Cognition	4.0597	0.59546					
Seeking Meaning	5.4087	1.20832	0.394**				
Possessing Meaning	5.2383	1.24617	0.548**	0.326**			
Intrinsic Motivation	4.0913	0.57677	0.632**	0.384**	0.632**		
Extrinsic Motivation	3.7225	0.51861	0.498**	0.303**	0.224**	0.398**	

**. Correlation is significant at the 0.01 level (2-tailed).

Table 3 The mediating effect test of positive cognition between sense of meaning in life and intrinsic motivation

Step	Path	Unstandardized Coefficients		Standardized Coefficients	t	p	Adjusted R^2
	X→Y	B	SD	β			
1	Seeking Meaning → Intrinsic Motivation	0.138^{***}	0.024	0.384	7.693	0.000	0.145
	Possessing Meaning → Intrinsic Motivation	0.292^{***}	0.019	0.632	15.085	0.000	0.397
2	Seeking Meaning → Positive Cognition	0.194^{***}	0.024	0.394	7.946	0.000	0.153
	Possessing Meaning → Positive Cognition	0.262^{***}	0.022	0.548	12.139	0.000	0.298
3	Seeking Meaning → Intrinsic Motivation	0.076^{***}	0.021	0.159	3.553	0.000	0.418
	Positive Cognition → Intrinsic Motivation	0.552^{***}	0.043	0.569	12.721	0.000	
	Possessing Meaning → Intrinsic Motivation	0.189^{***}	0.021	0.407	9.055	0.000	0.513
	Positive Cognition → Intrinsic Motivation	0.396^{***}	0.044	0.409	9.089	0.000	

* *p*<0.05 ** *p*<0.01 ****p*<0.001

Table 4 Bootstrap mediated effect test

Path	Effect	Effect Value	95% CI		SE	Conclusion
			LLCI	ULCI		
Seeking Meaning → Positive Cognition → Intrinsic Motivation	Toal Effect	0.183^{***}	0.136	0.230	0.024	Partial mediation
	Direct Effect	0.076^{***}	0.034	0.118	0.021	
	Indirect Effect	0.107	0.075	0.143	0.017	
Possessing Meaning → Positive Cognition → Intrinsic Motivation	Toal Effect	0.292^{***}	0.254	0.330	0.019	Partial mediation
	Direct Effect	0.189^{***}	0.148	0.229	0.021	
	Indirect Effect	0.104	0.070	0.141	0.018	

* *p*<0.05 ** *p*<0.01 *** *p*<0.001

Figure 1. Mediating effect pathway diagram

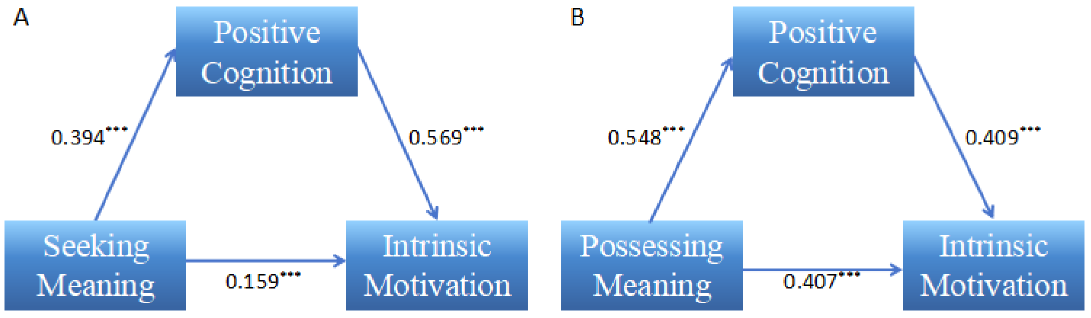

Table 5 The mediating effect test of positive cognition between the sense of meaning in life and extrinsic motivation

Step	Path	Unstandardized Coefficients		Standardized Coefficients	t	p	Adjusted R^2
	X→Y	B	SD	β			
1	Seeking Meaning → Extrinsic Motivation	0.130***	0.022	0.303	5.889	0.000	0.089
	Possessing Meaning → Extrinsic Motivation	0.093***	0.022	0.224	4.258	0.000	0.047
2	Seeking Meaning → Positive Cognition	0.194***	0.024	0.394	7.946	0.000	0.153
	Possessing Meaning → Positive Cognition	0.262***	0.022	0.548	12.139	0.000	0.298
3	Seeking Meaning → Extrinsic Motivation	0.054*	0.022	0.126	2.499	0.013	0.257
	Positive Cognition → Extrinsic Motivation	0.390***	0.044	0.448	8.860	0.000	
	Possessing Meaning → Extrinsic Motivation	-0.029	0.023	-0.070	-1.249	0.104	0.247
	Positive Cognition → Extrinsic Motivation	0.467***	0.049	0.536	9.584	0.000	

* $p<0.05$ ** $p<0.01$

Table 6 Bootstrap mediated effect test

Path	Effect	Effect Value	95% CI		SE	Conclusion
			LLCI	ULCI		
Seeking Meaning → Positive Cognition → Extrinsic Motivation	Toal Effect	0.130***	0.087	0.173	0.022	Partial mediation
	Direct Effect	0.054*	0.012	0.097	0.022	
	Indirect Effect	0.076	0.048	0.110	0.016	
Possessing Meaning → Positive Cognition → Extrinsic Motivation	Toal Effect	0.093***	0.050	0.136	0.022	Complete mediation
	Direct Effect	-0.029	-0.075	0.017	0.023	
	Indirect Effect	0.122	0.090	0.159	0.017	

* p<0.05 ** p<0.01 *** p<0.001

Figure 2. Mediating effect pathway diagram

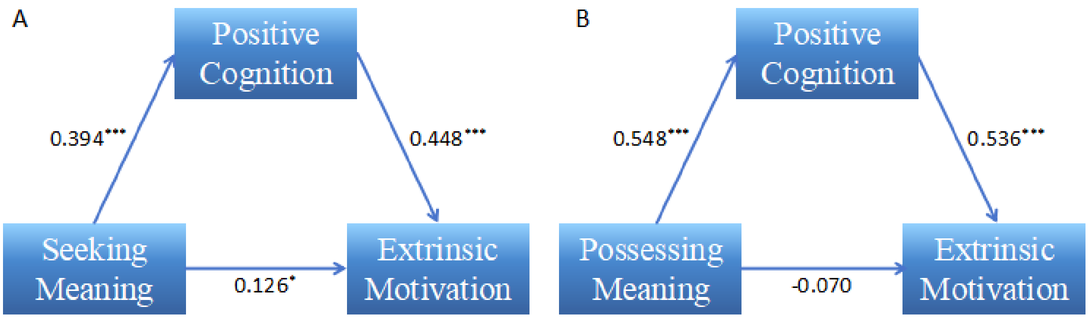

Click here to access/download;Figure;Figure 2.jpg

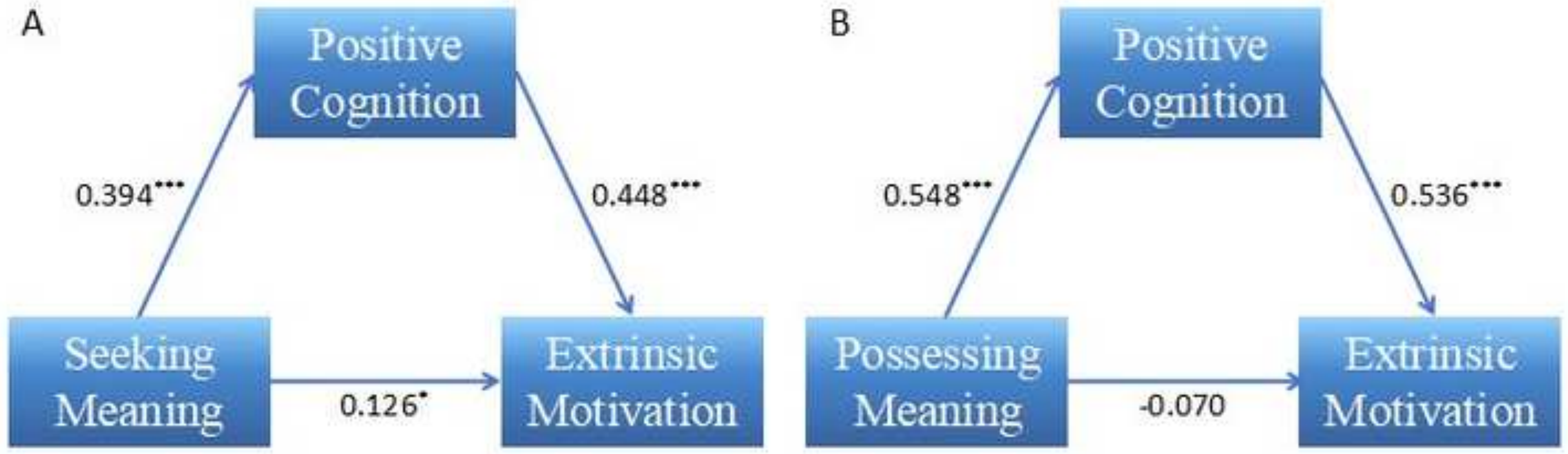

Click here to access/download;Figure;Figure 1.jpg

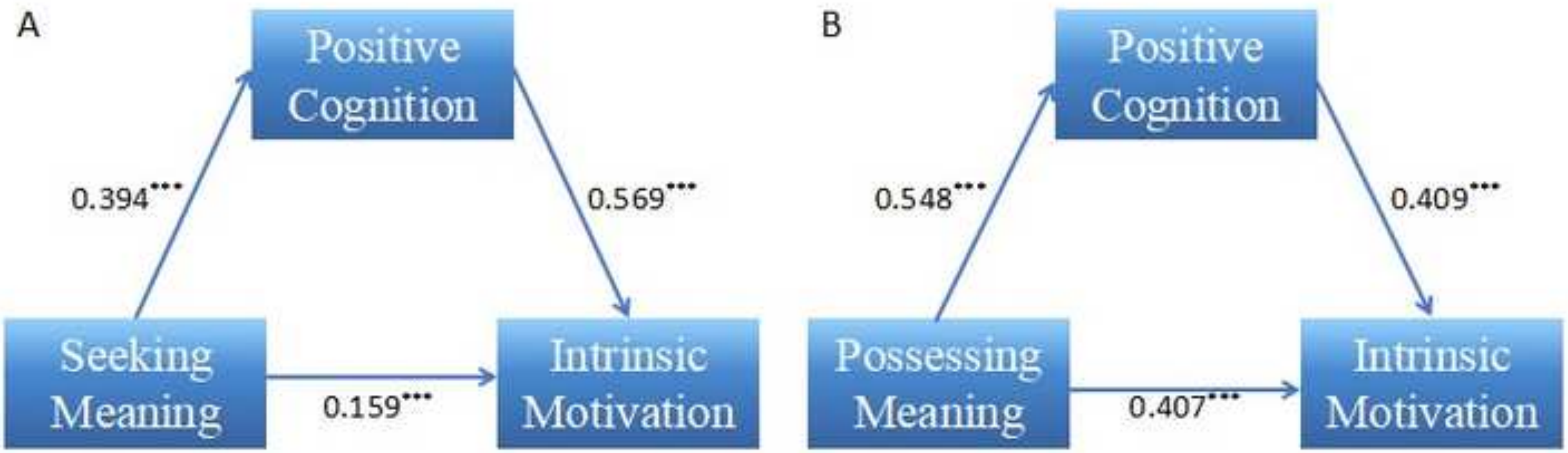

Supplement: Attachment — Submitted filename: commented PONE-D-25-25397_reviewer.pdf [file pone.0330447.s007.pdf]
