# Supplementary material for: Meaning in life, positive cognition, and learning motivation: A mediational analysis among Chinese college students
Source: PLoS One. 2025 Sep 12;20(9):e0330447. doi: 10.1371/journal.pone.0330447 (PMC12431252; doi:10.1371/journal.pone.0330447)
Supplement: Attachment — Submitted filename: Response to Reviewers.docx [file pone.0330447.s009.docx]

**Dear Editors,**

Thank you for giving us the opportunity to submit a revised version of our manuscript (Submission ID: PONE-D-25-25397). The revised manuscript entitled "**The Mediating Role of Positive Cognition in Connecting Life Meaning and Learning Motivation: A Cross-Sectional Study of Chinese College Students**" by Zhu et al., is available online for further consideration in **Plus one**. Our responses to reviewer’s comments are provided below on a point-to-point basis. We believe that the suggested changes have significantly improved the paper and hope that you will find our revised manuscript worthy of publication in your prestigious journal.

### ****Response to main points mentioned by academic editor****

***1. Please ensure that your manuscript meets PLOS ONE’s style requirements, including those for file naming.***

We thank the editor for this important guidance. We have conducted a thorough revision of the manuscript to ensure full compliance with PLOS ONE’s style requirements, using the official formatting samples (PLOSOne_formatting_sample_main _body.pdf and PLOSOne_formatting_sample_title_authors_affiliations.pdf) as a direct reference. Specifically:

Restructured the Title Page: We have corrected the author affiliations, removed extraneous symbols and footnotes from the author list, and ensured the corresponding author information is formatted correctly.

Standardized Headings: We have adjusted all section and subsection headings (e.g., Introduction, Methods, Results, Discussion) to match the specified font size, case, and numbering style.

Formatted Figures and Tables: We have ensured that all figure captions and table titles are placed correctly within the manuscript text and conform to the journal’s citation style (e.g., “Fig 1,” “Table 1”). The figures themselves have been removed from the manuscript file and will be uploaded as separate, high-resolution files.

We are confident that the revised manuscript now fully adheres to the journal’s formatting standards. We will also ensure all files are named according to the specified conventions upon resubmission.

***2. We note that the grant information you provided in the ‘Funding Information’ and ‘Financial Disclosure’ sections do not match. When you resubmit, please ensure that you provide the correct grant numbers for the awards you received for your study in the ‘Funding Information’ section.***

**We apologize for this oversight. This study was supported by grants from the Foundation of Basic Research and the Teaching Reform Project of the Army Medical University (Nos. Y2025W14 & 2022B12). We have been carefully checked and provide the correct grant numbers and funding information in the ‘Funding Information’ section of the submission system to ensure it matches the ‘Financial Disclosure’ statement.**

3. ***Thank you for uploading your study's underlying data set. Unfortunately, the repository you have noted in your Data Availability statement does not qualify as an acceptable data repository according to PLOS's standards.***

We have now deposited the minimal dataset necessary to replicate our study’s findings to figshare, a PLOS ONE recommended repository. The DOI for the dataset is provided in the “Supporting Information” section of our revised manuscript (S1 Appendix. Data collection <https://doi.org/10.6084/m9.figshare.29504990).> **Please refer to page 31, lines 654;**

***4. Please amend the manuscript submission data (via Edit Submission) to include author Zhiru Zhu.***

Author Zhi-ru Zhu has now been included by amending the manuscript submission data (via Edit Submission).

***5. Please amend your authorship list in your manuscript file to include author Zhi-ru Zhu.***

**We have amended the authorship list in the manuscript file to include Zhi-ru Zhu as the corresponding author.**

***6. Please remove your figures from within your manuscript file, leaving only the individual TIFF/EPS image files, uploaded separately. These will be automatically included in the reviewers’ PDF.***

**We have removed the figures from the manuscript file and upload them as separate, high-resolution TIFF/EPS files.**

**Response to Reviewers’ Comments**

We are grateful for the constructive comments and suggestions provided by the referees. All points raised were valid and fair. This paper is now revised in light of the referee’s suggestions.The referees’ concerns are addressed below on a point-to-point basis and recommended changes can be tracked in the revised paper (refer to either listed pages/lines, or sections highlighted in yellow).

**Response to reviewer 1**

Thank you for your valuable suggestions and your encouraging assessment of our work. Your comments on the manuscript’s structure and the presentation of our results to be especially helpful. In response to your feedback, we have reorganized the introduction into a more coherent structure, provided detailed descriptions of the instruments’ validity and reliability, and expanded our discussion to focus more on the mediation roles. We believe these changes have significantly improved the overall clarity of the paper and we appreciate your time and constructive comments.

***1. Introduction is too long. There should be a new part “Literature Review”***

As per the reviewer’s suggestion, the in**troduction has been shortened and a new part “Literature review” has been added to the** revised manuscript. **Please refer to page 4 to 9, lines 79 to 199;**

2. ***Theoretical fundations (SDT) should be highlighted in a section in Literature Review.***

**Thank you for this valuable advice. We have added a new subsection “2.1 Theoretical Framework Construction” within the Literature Review. In this section, we explicitly highlight Self-Determination Theory (SDT) and integrate it with the Broaden-and-Build Theory to provide a stronger theoretical foundation for our study. We have also included a theoretical model diagram (Fig 1) to visually represent our framework. Please refer to page 4 to 5, lines 80 to 101;**

***3. The gap or the need for the study should be rewritten. The present version is not well developed in saying this point.***

**We appreciate this important point. We have rewritten and expanded the section “2.5 The Current Study” to more clearly and robustly articulate the research gaps and the necessity of our investigation. We now explicitly state the lack of research on positive cognition as a mediator and the need for a theoretically integrated model. Please refer to page 8, lines 175 to 186;**

***4. The instruments should be added some descriptions of validity and reliablity.***

**As per the reviewer’s suggestion, we have added detailed descriptions of the validity and reliability for all instruments used. In section “3.2 Instruments” , we now report Cronbach’s alpha coefficients, confirmatory factor analysis (CFA) fit indices, Average Variance Extracted (AVE), and Composite Reliability (CR) for each scale. Please refer to page 10 to 12, lines 223 to 269.**

***5. The findings should be given more space for mediation roles report. Correspondingly, the discussion part should say more about this report.***

**We thank the reviewer for this suggestion. We have expanded the results and discussion sections to focus more on the mediation analysis. The “4.3 Mediation analysis” section (pages 14-20) now provides detailed tables and path diagrams for the mediation effects. Furthermore, the discussion section, particularly “5.2 The core mechanism: How positive cognition translates meaning into motivation” (page 21), is now dedicated to a thorough interpretation of these mediation findings. Please refer to page 14 to 20, and 21**

**Response to reviewer 2**

Thank you for your valuable suggestions and your encouraging assessment of our work. Your comments on the manuscript’s theoretical foundation, methodological rigor, and the need for clearer reporting of results were especially helpful. In response to your feedback, we have expanded the theoretical justification for positive cognition as the mediator by integrating frameworks such as Self-Determination Theory and Broaden-and-Build Theory, enhanced the mediation analysis using more robust techniques like the PROCESS macro, revised the discussion to avoid causal overstatements and include detailed effect size clarifications, and addressed issues with citations, terminology consistency, and supplementary materials. We believe these changes have significantly strengthened the scientific rigor and clarity of the paper, and we appreciate your time and constructive comments.

***1.This study explores the mediating role of positive The conceptual rationale for using positive cognition as the sole mediator is underdeveloped. Why was positive cognition chosen over other potentially relevant mediators such as self-efficacy, resilience, or optimism? The manuscript lacks a detailed theoretical model illustrating the hypothesized relationships (figures). Inclusion of a theoretical model earlier would enhance clarity. I suggest to expand the theoretical justification for the choice of mediator. Consider integrating a broader theoretical lens (e.g.,Self-Determination Theory, Broaden-and-Build Theory) to justify the mechanisms. The following article may help how to structure article and report methods. Du, M. & Ashraf, M. A. (2025). Antecedents of environmental protection participation intentions among Chinese adolescents: The perspectives of rational choice, moral norms, and supporting environment. British Educational Research Journal, 00, 1–25. https://doi.org/10.1002/berj.41682.***

We thank you for this critical comment. We have addressed your concerns in several ways. Firstly, we have added a new section “2.1 Theoretical Framework Construction” (page 4) and significantly expanded “2.4 The mediating role of positive cognition” (page 6) to provide a much stronger theoretical rationale, integrating Self-Determination Theory and the Broaden-and-Build Theory. Secondly, we have explicitly justified our choice of positive cognition over other potential mediators. Thirdly, we have added a new theoretical model diagram (Fig 1, page 5) to clearly illustrate the hypothesized relationships. We also thank you for the recommended article (Du & Ashraf, 2025), which we have now cited [2] and learned from in structuring our arguments. **Please refer to page 4 to 6,line 80 to 101 and 130 to 174**

***2.The Learning Motivation Questionnaire’s extrinsic motivation subscale shows poor construct validity (AVE = 0.28). This raises concerns about the adequacy of this scale for capturing extrinsic motivation.***

**Thank you for your valuable comment regarding the construct validity of the extrinsic motivation subscale in the learning motivation questionnaire, particularly the concern about the low average variance extracted (AVE = 0.28). We acknowledge this limitation but would like to provide a comprehensive explanation for the continued use of this subscale based on multiple psychometric indicators.**

**Firstly, the composite reliability (CR) of the extrinsic motivation subscale is 0.72, which exceeds the widely accepted threshold of 0.7, indicating strong internal consistency among the items (Hair et al., 2010).**

**Secondly, discriminant validity is adequately established through two rigorous tests: all Pearson correlations between factors are smaller than the square root of their corresponding AVE (satisfying the Fornell-Larcker criterion), and all heterotrait-monotrait ratios (HTMT) are below 0.85, demonstrating that the scale can effectively distinguish between different constructs (Henseler et al., 2015),as shown in Table HTMT.**

| **Table HTMT** | | | | | |
| --- | --- | --- | --- | --- | --- |
|  | Positive Cognition | Seeking Meaning | Possessing Meaning | Intrinsic Motivation | Extrinsic Motivation |
| Positive Cognition | - |  |  |  |  |
| Seeking Meaning | 0.452 | - |  |  |  |
| Possessing Meaning | 0.603 | 0.382 | - |  |  |
| Intrinsic Motivation | 0.702 | 0.446 | 0.703 | - |  |
| Extrinsic Motivation | 0.672 | 0.401 | 0.464 | 0.715 | - |
| All HTMT are less than 0.85. | | | | | |

**Notably, scholars have emphasized that AVE is not the sole or absolute criterion for evaluating construct validity. When composite reliability is satisfactory and discriminant validity is well-established, a lower AVE does not necessarily invalidate the scale (Hair et al., 2006). For instance, Donche et al. (2010) found that some subscales of the Learning Strategies and Motivation Questionnaire (LEMO) had AVE values below 0.5 but were still deemed valid due to strong composite reliability and discriminant validity, which aligns with our case.**

**Given these considerations, we believe the extrinsic motivation subscale remains appropriate for the current study, and we have clarified these points in the revised manuscript to address potential concerns. We appreciate your critical feedback, which has helped us strengthen the rigor of our psychometric discussions.**

Reference:

1. Hair, J. F., Black, W. C., Babin, B. J., & Anderson, R. E. (2010). *Multivariate data analysis*. Pearson.
2. Henseler, J., Ringle, C. M., & Sarstedt, M. (2015). A new criterion for assessing discriminant validity in variance-based structural equation modeling. *Journal of the Academy of Marketing Science*, 43(1), 115-135.
3. Hair, J. F., Ringle, C. M., & Sarstedt, M. (2006). PLS-SEM: Indeed a silver bullet. *Journal of Marketing Theory and Practice*, 14(1), 139-152.
4. Donche, V., Van Petegem, P., Van de Mosselaer, P., & Vermunt, J. D. (2010). Development and validation of the Learning Strategies and Motivation Questionnaire (LEMO). *Studies in Higher Education*, 35(3), 319-338.

***In addition, Positive Cognition is operationalized through a single subscale (positive attention), which might not fully represent the broader construct of positive cognition.***

Thank you for your insightful comment regarding the operationalization of Positive Cognition through the single subscale of positive attention. We acknowledge the concern that this may not fully capture the breadth of the construct, and we appreciate the opportunity to clarify our rationale.

The selection of positive attention as a key indicator of Positive Cognition is grounded in theoretical and empirical precedents. Conceptually, positive attention—defined as the deliberate focus on positive stimuli or aspects of experience—serves as a foundational component of positive cognitive processes (Fredrickson, 2001). It is considered a core mechanism through which individuals allocate cognitive resources to positive information, forming the basis for downstream processes such as positive interpretation or expectation (Gable & Harmon-Jones, 2008). Empirically, studies have demonstrated that positive attention robustly predicts broader positive cognitive tendencies: for instance, individuals who habitually attend to positive cues show stronger positive reappraisal abilities (Bryant, 2003) and higher resilience in cognitive evaluations of stressors (Cohn et al., 2009).

Moreover, positive attention has been validated as a reliable and measurable indicator in applied research. Its operationalization via self-report or behavioral tasks (e.g., dot-probe paradigms) shows good psychometric properties, making it suitable for large-scale or field-based studies (Isaacowitz, 2005). Notably, several studies investigating positive cognition in educational or clinical contexts have successfully used positive attention as a proxy, finding significant associations with well-being, academic performance, and emotional regulation (e.g., Garland et al., 2010; Rowe et al., 2007). These precedents support its utility as a practical and meaningful indicator of Positive Cognition, particularly in studies prioritizing feasibility without sacrificing theoretical relevance.

In the "Discussion-Limitations" section, we have added a note acknowledging that this operationalization may underrepresent the multidimensionality of Positive Cognition, which inherently includes positive interpretation, future-oriented positive expectations, and other facets (Diener et al., 2018; Seligman & Csikszentmihalyi, 2000). We suggest that future research adopt comprehensive measures (e.g., the Positive Cognitive Style Questionnaire, which assesses attention, interpretation, and expectation; Peterson et al., 1988) to capture the full scope of the construct and enhance generalizability.

Reference:

1. Fredrickson, B. L. (2001). The role of positive emotions in positive psychology: The broaden-and-build theory of positive emotions. *American Psychologist, 56*(3), 218–226.
2. Gable, P. A., & Harmon-Jones, E. (2008). Approach-motivated positive affect reduces breadth of attention. *Psychological Science, 19*(4), 326–332.
3. Bryant, F. B. (2003). Savoring: A new model of positive experience. Lawrence Erlbaum Associates.
4. Cohn, M. A., Fredrickson, B. L., Brown, S. L., Mikels, J. A., & Conway, A. M. (2009). Happiness unpacked: Positive emotions increase life satisfaction by building resilience. *Emotion, 9*(3), 361–368.
5. Isaacowitz, D. M. (2005). Use of gaze for real-time mood regulation: Effects of age and attentional functioning. *Psychology and Aging, 20*(4), 588–599.
6. Garland, E. L., Fredrickson, B. L., Kring, A. M., Johnson, D. P., Meyer, P. S., & Penn, D. L. (2010). Upward spirals of positive emotions counter downward spirals of negativity: Insights from the broaden-and-build theory and affective neuroscience. *Current Directions in Psychological Science, 19*(2), 87–91.
7. Rowe, G., Hirsh, J. B., & Anderson, A. K. (2007). Positive affect increases the breadth of attentional selection. *Proceedings of the National Academy of Sciences, 104*(1), 383–388.
8. Diener, E., Lucas, R. E., & Scollon, C. N. (2018). Beyond the hedonic treadmill: Revisiting well-being dynamics. *Current Directions in Psychological Science, 27*(3), 175–180.
9. Seligman, M. E., & Csikszentmihalyi, M. (2000). Positive psychology: An introduction. *American Psychologist, 55*(1), 5–14.
10. Peterson, C., Semmel, A., von Baeyer, C., Abramson, L. Y., Metalsky, G. I., & Seligman, M. E. (1988). The Attributional Style Questionnaire. *Cognitive Therapy and Research, 12*(3), 357–360.

***3.Although bootstrapping was used, no justification was provided for the sample size’s adequacy in mediation analysis. Hierarchical regression analysis is appropriate, but more sophisticated mediation analysis using SEM or PROCESS macro would provide more robust insights.***

Thank you for your valuable suggestions regarding the mediation analysis. We appreciate your reminder about justifying the sample size adequacy and using more sophisticated methods, which has helped us improve the rigor of our analysis.Regarding the sample size for bootstrapping-based mediation analysis, we have supplemented relevant justifications. Based on Fritz & MacKinnon (2007), bootstrap mediation analysis requires sample sizes of ≥546 for small effects (e.g., a=0.14, b=0.14) and ≥71 for medium effects (e.g., a=0.39, b=0.39). Our sample size of 345 exceeds the requirement for medium effects and provides ≥80% power for most cases where at least one path (a or b) is medium-sized (≥0.26). Although limitations exist if both paths are very small (<0.14), overall, 345 is adequate for bootstrap-based mediation in typical social science research with expected path coefficients ≥0.20.

In response to your suggestion about more sophisticated mediation analysis, we would like to clarify that besides hierarchical regression analysis (proposed by Wen Zhonglin), we also conducted mediation analyses using Hayes' PROCESS macro (Model 4) in SPSS 24.0, which is a widely recognized and robust method for examining mediation effects. This approach allows for more rigorous testing of the mediating role of positive cognition between a sense of meaning in life and intrinsic motivation, complementing the hierarchical regression results.

We have incorporated these details into the revised manuscript to enhance the transparency and robustness of our mediation analysis. **Please refer to page 14 to 15,line 285 to 300**

Reference:

1. Fritz MS, MacKinnon DP. Required sample size to detect the mediated effect. Psychol Sci. 2007;18(3):233-9.

***4.The discussion sometimes overstates the findings, particularly regarding causality (e.g., phrases like “enhancing life meaning fosters...” imply causation, which is inappropriate for cross-sectional data). The authors mention that 58.5% of the effect is mediated but fail to clarify whether this is a proportion of the standardized total effect or unstandardized. Figure 1 and Figure 2 are insufficiently detailed (e.g., missing p-values, standard errors).***

We thank the reviewer for this important feedback.We have carefully revised the manuscript, especially the Discussion section, to use more cautious language (e.g., “is associated with” instead of “fosters”) to avoid implying causality from our cross-sectional data.We have clarified that the proportion of the mediated effect was calculated using unstandardized effects (e.g., “The proportion of the mediating effect to the total effect was 0.107/0.183 × 100% = 58.5%”, page 16).Our figures have been revised. The original theoretical model figures are now combined into one clear diagram (Fig 1). The results path diagrams (now Fig 3 and Fig 4, pages 17 & 20) have been updated to include standardized path coefficients and asterisks indicating p-values, providing much more detail.**Please refer to Fig 1,3 & 4,page 5,18 & 21, line 101,340&386.**

***5.Revise the manuscript for conciseness and consistency in terminology. Consider reorganizing or trimming redundant sections (especially in the Discussion and Limitations).***

We appreciate your suggestion to enhance the manuscript’s conciseness and consistency. In response, we have shortened the Introduction by approximately 30% (now limited to one page), reorganized the Discussion to eliminate redundancies (e.g., merging overlapping interpretations in sections 5.1 and 5.2), and trimmed the Limitations section by focusing on key constraints while removing repetitive phrasing. We have also ensured terminology consistency (e.g., uniformly using “meaning in life” instead of varying synonyms). These revisions have made the manuscript more streamlined and reader-friendly. **Please refer to pages 3 to 4 (Introduction), 21 to 23 (Discussion), and the Limitations section (pages 25 to 26).**

***6.Several citations are from lesser-known journals or in Chinese; efforts should be made to include more international, high-impact sources if possible. The literature review is limited in its scope and in-depth analysis of the topic. Massive literature is available on meaning and learning motivation in Chinese and other nation's context.***

We are very grateful to you for these specific and constructive suggestions. We especially appreciate the recommendation of the articles by Feng et al. (2025) and Cheng et al. (2021), which we agree are highly relevant to our study.

In response to your valuable feedback, we have undertaken a comprehensive revision of the literature review:

Integration of Recommended and High-Impact Sources: We have now cited the insightful studies you recommended by Feng et al. (2025) and Cheng et al. (2021) to enrich our discussion. Furthermore, we have expanded our reference list with numerous articles from leading international journals to strengthen our theoretical framework, including works from Nature Reviews Psychology (Gagné et al., 2022), the Journal of Personality and Social Psychology (Slemp et al., 2024), Psychological Bulletin (Howard et al., 2017), American Psychologist (Ryan & Deci, 2000; Fredrickson, 2001), and the Annual Review of Psychology (King & Hicks, 2021).

Enhanced Scope and In-depth Analysis: The updated literature review now provides a more in-depth analysis, drawing stronger connections between core theories (e.g., Self-Determination Theory, Broaden-and-Build Theory) and recent empirical findings. By incorporating a wider range of literature, we have better contextualized our research within both the Chinese educational landscape (e.g., Wei et al., 2024; Liu et al., 2025) and the broader international conversation on this topic.

Justification for Context-Specific Citations: We have retained a few key Chinese-language citations (e.g., Liu & Gan, 2010; Dai et al., 2015) as they pertain directly to the validation of the measurement scales used in our study. We believe this is essential for methodological transparency and reproducibility within the specific cultural context.**Please refer to pages 3-9, lines 49-199, and the updated Reference list.**

***7. Why is Glossary added to the article? There is no need of glossary in academic journal article.***

We apologize for this error in the previous version. The glossary has been removed from the revised manuscript.

***8. The abstract is too dense and technical; simplify and clarify mediation results for a broader audience.***

The abstract has been completely rewritten to be more accessible, concise, and clear. It now focuses on the core findings and their practical implications in a way that is understandable to a broader audience. **Please refer to page 3.**

***9. Provide the questionnaire and other necessary data in supplementary materials.***

As requested, we have created a “Supporting Information” section at the end of the manuscript. This section includes a public link (figshare) to our minimal dataset (S1 Appendix) and a reference to the questionnaires used in the study (S2 Appendix). **Please refer to page 31.**
